# An *In Situ*, Individual-Based Approach to Quantify Connectivity of Marine Fish: Ontogenetic Movements and Residency of Lingcod

**DOI:** 10.1371/journal.pone.0014267

**Published:** 2010-12-13

**Authors:** Mary Anne Bishop, Brad F. Reynolds, Sean P. Powers

**Affiliations:** 1 Prince William Sound Science Center, Cordova, Alaska, United States of America; 2 Dauphin Island Sea Lab, Department of Marine Sciences, University of South Alabama and Center for Ecosystem Based Fisheries Management, Dauphin Island, Alabama, United States of America; National Oceanic and Atmospheric Administration/National Marine Fisheries Service/Southwest Fisheries Science Center, United States of America

## Abstract

As modern fishery assessments change in an effort to be more accurate and encompass the range of potential ecosystem interactions, critical information on the ecology of species including life history, intra and inter-specific competitive interactions and habitat requirements must be added to the standard fishery-dependent and independent data sets. One species whose movements and habitat associations greatly affects exploitation patterns is lingcod, *Ophiodon elongatus*, which support an economically important fishery along the coastal waters of the Pacific Coast of North America. High site fidelity and limited movements within nearshore areas are hypothesized to have resulted in high catchability, a major factor that has contributed to overfished stocks. Thus, assessing the level of movement and connectivity among lingcod subpopulations inhabiting nearshore habitats is a prerequisite to determining the condition of lingcod stocks. We used the Pacific Ocean Shelf Tracking (POST) Project acoustic receiver array in Alaska's Prince William Sound to monitor movements and residency of 21 acoustic-tagged lingcod for up to 16 months. Eight of sixteen lingcod (50%) initially aged at 2.5- to 3.5- years-old dispersed from their tag site. Dispersal was highly seasonal, occurring in two, five-week periods from mid-December through January and from mid-April through May. Dispersal in winter may be related to sexually immature lingcod or newly-mature male lingcod being displaced by territorial males. Spring dispersal may be indicative of the onset of migratory behavior where lingcod move out into Prince William Sound and possibly the offshore waters of the Gulf of Alaska. Our results reveal a pattern of ontogenetic dispersal as lingcod approach 4-years-old and exceed 50 cm total length. The large proportion of tagged fish migrating out of Port Gravina, their tagging site, reflects a high level of connectivity among Prince William Sound subpopulations. Our results also support the hypotheses that these subpopulations may be highly susceptible to overfishing because most fish show long residence times.

## Introduction

Movements of mobile fish can influence both ecological and fisheries interactions on multiple spatial scales. Large-scale (100's of km) movements occur primarily via egg and larval dispersal in the early life stages of most marine invertebrates and fishes and have profound effects on fishery stock dynamics because of the high potential for connectivity [1]. Lingcod *Ophiodon elongatus* is an exception to this generic paradigm because there is no egg dispersal, eggs are deposited at nest sites, and larvae are relatively large when in the plankton. After larval settlement, movements are limited for some period of time until animals attain larger sizes [2].

Smaller or regional scale (m–km's) connectivity patterns of juvenile and adult fish are influenced by a combination of morphological, behavioral and environmental variables. Although regional and local (m's) scale movements have relatively minor impacts on stock ranges, movements on these scales can greatly influence ecological and fisheries interactions. For example, the outcome of competitive and predator-prey interactions can be modified by movements of animals, particularly around structured habitats [3]. Similarly, fisheries interaction can be influenced by movement patterns with fish showing high site fidelity and limited movement being easier to exploit by technologically advanced fishers (e.g. GPS and sonar that locates bottom structure). Repeated use of the same areas by fish can dramatically increase catchability via reduced unit effort to find a fish, which may not be predicted by fishery models and thus could lead to higher exploitation levels [4].

Until recently, investigations of regional and local patterns of movements were limited to ranges of direct visual observations or endpoints from mark and recapture studies. Recent advances in the miniaturization and cost effectiveness of acoustic telemetry have increased (e.g. [5]) the capacity of marine scientists to investigate patterns of site fidelity, residency and home ranges as well as describe potential migration routes and connectivity patterns. Over the last two decades, tracking of mobile fauna was restricted to manual monitoring via vessel based, hydrophone surveys. Animals acoustically tagged could be followed for some immediate time period after implantation and potentially relocated and followed again. More recently, the availability of autonomous recording hydrophones has allowed continuous *in situ* monitoring of key habitats and migration routes. When properly calibrated and maintained, such autonomous moorings allow inference to be drawn on fish behavior from both presence and absence of detections [6]. Here, we utilize an autonomous array of fixed hydrophones to examine behavior of a heavily exploited marine fish, lingcod.

Lingcod are found only along the coastal waters of the Pacific Coast of North America [2] and support an important recreational and commercial fishery. Currently, lingcod is a species of critical concern to fisheries managers throughout the Pacific Coast because of the combined factors of low annual productivity [7] and susceptibility to overfishing are a result of their high site residency and association with the nearshore zone [8]–[10]. In Canada's Strait of Georgia, the lingcod commercial fishery has been closed since 1990 [11] while in Washington, Oregon, and California lingcod was declared an overfished species between 1999 and 2005 [12].

Assessing the level of connectivity among lingcod subpopulations is a prerequisite to determining the condition of lingcod stocks. Until recently, studies have relied on mark and recapture data to reveal the complexity of lingcod movements. In the Strait of Georgia, lingcod recaptures showed females dispersing more often and moving longer distances than males [13]. Two studies in the Strait of Juan de la Fuca, documented a high percentage of migratory behavior in lingcod. Both studies defined migratory lingcod as recaptures >8.1 km from the initial tag site. The first study tagged fish in the eastern portion of the Strait and found 50% of recaptured lingcod were migratory, with no evidence of sex differences [14]. The second study tagged lingcod in the western Strait and verified migratory behavior in 19% of the recaptured lingcod. They observed that males were more likely to migrate than females [8]. In the Gulf of Alaska, recoveries of lingcod initially tagged in southeast Alaska indicate that some female lingcod make long-distance movements of up to 775 km while the longest movement documented for a male was 106 km (www.cf.adfg.state.ak.us/region1/finfish/grndfish/lingcod/lresearch.php).

Acoustic transmitters make it possible to monitor fish movements both across large distances [6] and in structurally complex habitats like those found in nearshore areas [15]. Until recently, the only study of acoustic-tagged lingcod in Alaska was conducted at the Edgecumbe Pinnacles Marine Reserve in southeast Alaska. Tagged fish frequently left the reserve, but returned following short absences [10], [16]. In 2007 we used acoustic telemetry in southcentral Alaska's Prince William Sound to document residency and movements of lingcod and rockfish (primarily copper rockfish *Sebastes caurinus*) during summer months. We noted that 2- and 3-year-old lingcod appeared to move into reef habitats in mid-summer and were still present in early fall when the study was concluded [17]. In October 2008, the first long-term, autonomous acoustic telemetry array was installed in Prince William Sound as part of the Pacific Ocean Shelf Tracking (POST) Project. Here we present data on residency and movements by lingcod tagged in the vicinity of the array and monitored over 16 months. The objectives of this study were to: 1) quantify residency of lingcod, 2) describe lingcod movement patterns in the nearshore zone, and 3) determine if there are ontogenetic differences in movements and residency.

## Results

**Figure 1:**
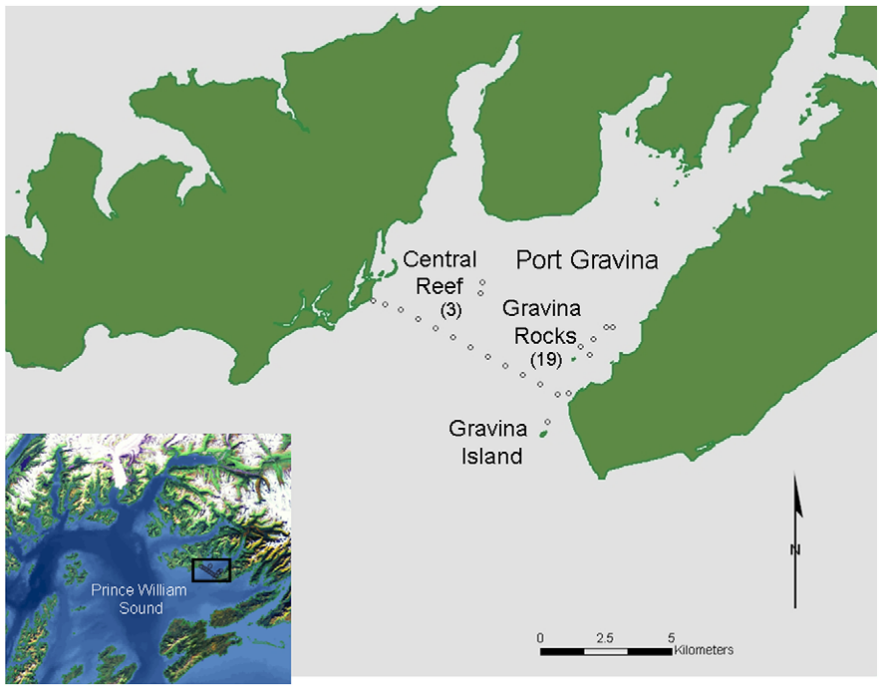
Location of lingcod tagging and acoustic monitoring areas in northeast Prince William Sound, Alaska. Fish were tagged at Gravina Rocks (n = 19 fish) and central reef areas (n = 3 fish). Each unfilled circle represents a VR2 or VR3 receiver.

Acoustic transmitters were implanted at Port Gravina in fourteen and eight lingcod during October 2008 and late July/mid August 2009, respectively (Table 1, Figure 1). Total length (TL) of tagged fish ranged from 46.5 to 125 cm. Total length for lingcod captured in summer 2009 was significantly larger than fall 2008 lingcod (t-test *P*<0.02). Based on TL at capture, ages for 73% of the 22 tagged lingcod were estimated at 2.5- and 3.5-years-old. Seven fish were estimated at 2.5-years (TL range: 46.5–47.9 cm) and four fish at 3.5-years (TL range: 54.0–55.0 cm), while five fish with intermediate TL (range: 50.5–51.4 cm) were not assigned an exact age. A 68.7 cm TL lingcod with an estimated age of 4.5-years at the time of its October 2008 capture was later preyed upon. We tagged four females and one male adult lingcod that were ≥6-years and ranged in size from 90 to125 cm TL. One of the adult females was detected infrequently and therefore excluded from further analyses.

**Table 1:** Lingcod total length (cm) by Port Gravina capture location and date.

Location	Month/Year	Number Tagged	Total Length (cm) 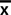 ±SE	Total Length (cm) Range
Central Reef	Oct 2008	3^a^	57.8±5.6	50.6–68.7
Gravina Rocks	Oct 2008	11^b^	53.6±5.3	46.5–106.0
Gravina Rocks	Jul/Aug 2009	8	78.8±10.1	51.0–125.0

a One fish preyed upon, May 2009.

b One fish not included in residency and movement analyses due to minimal detections.

Tagged fish resided at their capture area on average 98.5%±5% of the days monitored. Mean consecutive days of residency for fall 2008 tag cohorts was 279±35 d (range: 125–485; *n* = 13) and for the summer 2009 tag cohort 139±21 d (range: 38–210, *n* = 8). Five (38%) of the fall 2008 and six (75%) of the summer 2009 tag cohorts remained at their tag and release site at the end of this study. With the exception of one predation event, no other mortalities were detected.

Seventeen of the twenty-one monitored lingcod were absent ≥1 d from their tagging area while four lingcod appear to be sedentary with no absences detected. Temporary absences followed by a return to the tag area had a mode length of 1 d (max = 27 d, *n* = 42). Average duration of absences did not differ between individuals (ANOVA, df = 1, F = 0.11, *P = *0.74, *n* = 11). Lingcod tagged in fall 2008 averaged only 2.2±0.9 temporary absences (max = 9, *n* = 13 fish) over 16 months of monitoring. Within the summer 2009 cohort, there was a trend for the four large adult lingcod to move out of the study area (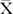 = 2.75 absences) more often than the smaller lingcod (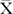 = 0.5 absences) although the difference was not significant (t-test, *P = *0.15). There were interannual differences in absences. None of the fall 2008 tag cohorts were absent from October 2008, when first tagged, through the beginning of March 2009. In contrast, absences were detected the following winter in lingcod from both tag cohorts for all months except November 2009 and peak numbers of absent lingcod were recorded in December 2009 (*n* = 4 fish) and January 2010 (*n* = 5 fish).

While it is unknown where most lingcod moved to during their absences, a 125 cm TL female was detected moving south from Gravina Rocks on 7 February 2010 with a final detection occurring at Gravina Island, our southernmost receiver. Thirty-six hours later, on 8 February, this female was detected again at Gravina Island. She continued moving north, returning to her Gravina Rocks tagging area that same day. The following day, she made a round trip from Gravina Rocks to the Port Gravina curtain.

**Figure 2:**
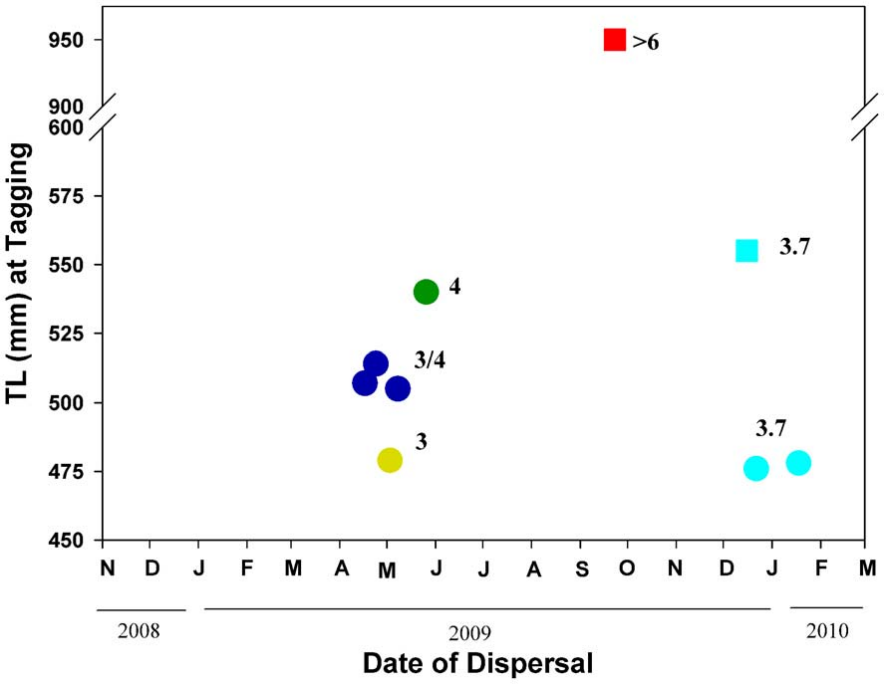
Total length (TL) at capture and date of dispersal from tagging area for acoustic-tagged lingcod. Number  =  estimated age at dispersal. Circles  =  tagged fall 2008; squares  =  tagged summer 2009. Sex could not be determined for fish <600 mm. The 950 mm lingcod departing in September 2009 was an adult male.

Almost one-half of the tagged lingcod dispersed from their initial tagging site including 7 of 12 lingcod tagged in fall 2008, and 2 of the 8 fish tagged in summer 2009 (Figure 2). Except for a >6-year-old adult male, all other lingcod that dispersed were 2.5- and 3.5-years-old at tagging. Dispersal phenology was seasonal with eight of the nine lingcod leaving their tagging site between 17 April and 23 May (*n* = 5) or between 16 December and 18 January (*n* = 3). Lingcod dispersing in spring 2009 were significantly larger (TL) at tagging than their non-dispersing tag cohorts (t-test, *P* = 0.01).

**Figure 3:**
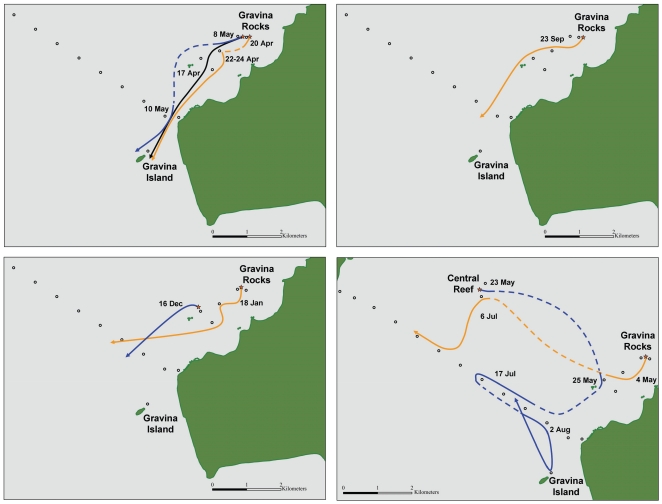
Schematic diagram of dispersal movements of eight lingcod from Port Gravina tagging areas. a) Lingcod dispersing out of Port Gravina, spring 2009. b) Lingcod dispersing from their tagging site in spring 2009 and remaining in Port Gravina. c) Lingcod dispersing out of Port Gravina, fall 2009. d) Lingcod dispersing out of Port Gravina, winter 2009–2010. Colored lines denote individual lingcod and arrows denote direction of travel. Dashed lines indicate absences >1 d without detection.

Prior to their departure, behavior by dispersing lingcod tended to be similar. Six of the nine fish had been sedentary with no previous absences from their tagging area detected. Another two of the nine lingcod had been previously absent, including for 1 to 2 d in the week before their departure. Dispersal movements tended to be southerly and relatively rapid with most fish detected at multiple receivers over a <24 h period before disappearing (Figure 3). Final detections for the majority of the dispersing lingcod were either at the Port Gravina curtain (3 of 9) or past the curtain and at Gravina Island, our southernmost receiver (3 of 9), suggesting that these fish left Port Gravina. At the time of dispersal, three lingcod were not detected at the Port Gravina curtain or at Gravina Island. Two of the three lingcod were detected at the Port Gravina curtain approximately two months later suggesting they are still in Port Gravina (Figure 3b).

## Discussion

Our array of autonomous hydrophones revealed important aspects of movement and behavior of lingcod in Prince William Sound and, more broadly, demonstrates the efficacy of maintaining a fixed array of hydrophones. Not surprisingly, our individual-based approach revealed significant individual variability in migration timing, and residency time. By combining these individual results a pattern of ontogenetic dispersal emerged for young (ages 2–4) lingcod as they approach 4 years in age and exceed 50 cm TL. Lingcod that dispersed in December and January each had an estimated age of 3.7-years. Within the fall 2008 tag cohort, only one of seven smallest lingcod (47.9 cm TL) dispersed in spring 2009 at the age of 3.0-years. In contrast, three of four lingcod in the fall 2008 tag cohort that were slightly larger (TL range: 50.5–51.4 cm) dispersed in spring 2009 suggesting that they were 4-years-old.

Dispersal was highly seasonal occurring primarily during two, five-week periods: mid-December through January and mid-April through May. Dispersal in winter may be related to sexually immature lingcod or newly-mature male lingcod being displaced by larger, territorial males. In Alaska, lingcod spawn from January through March [18], but males establish territories as early as November [19]. In British Columbia, movements of larger, mature lingcod into spawning habitat during January and February has been associated with a decrease in lingcod densities as well as fewer small (<50 cm TL) lingcod [20]. Further monitoring will elucidate if the Port Gravina lingcod displaced by breeders return following the departure of nest-guarding males.

Dispersal in spring may be indicative of the onset of migratory behavior where lingcod move out into Prince William Sound and possibly the offshore waters of the Gulf of Alaska. In the western Strait of Juan de Fuca lingcod densities decline from April to August as a significant number of both male and female adult lingcod migrate out from their nearshore habitats. There, larger lingcod tend to migrate further and to offshore, open waters [21].

Our research demonstrates that acoustic arrays can be an effective means of obtaining precise information on the timing and direction of lingcod dispersal. Based on the detections, we were able to determine that six fish migrated out of Port Gravina. For three of these fish, their migration route followed the shoreline and their final detection occurred at our southernmost receiver, positioned 1.2 km south of the Port Gravina curtain. Lingcod departing on 16 December and 18 January migrated further from the shoreline and were detected at the Port Gravina curtain but not at the one receiver south of the curtain. Two of three lingcod not detected at the Port Gravina curtain at the time of dispersal were later detected within Port Gravina, suggesting that detection at the curtain during a dispersal movement is indicative of leaving Port Gravina. Unexpectedly, our acoustic array provided accurate information on the timing and direction of a lingcod being preyed upon and removed from the study area. We were able to conclude that the predator was likely a marine mammal based on its speed, and in this case, the depth of the predator as the lingcod had a pressure sensor tag implanted.

Of the four large adults tagged, only the male dispersed from the study area. This male was exceptionally large; his 950 cm TL being equal to the maximum TL recorded for male lingcod in southeast Alaska [18]. In the Strait of Georgia, large nesting males are associated with deeper waters >40 m, while smaller males are associated with nesting areas in waters 5 to 25 m deep [2]. Depths around the Gravina Rocks pinnacles are relatively shallow (10–20 m). Given the large size of this male, his movement out of Port Gravina in mid-September may indicate movement to a deeper spawning site. Our sample of older female lingcod was small (three females) and monitoring concluded on 23 February, before the end of the breeding season. While two of the older females were relatively sedentary during the August to February monitoring period, the third female that left in early February for two days before returning, probably left to spawn. A similar, rapid movement was recorded in California when a lingcod believed to be a female moved 16 km in January before returning to her original tagging site [22].

Of the smaller lingcod that we tagged (<60 cm TL), only males would have potentially become sexually mature during the course of this 16-month study. Minimum TL at maturity for female lingcod in southeast Alaska is 68 cm [18], while our largest lingcod <60 cm was only 56 cm. Size at maturity for male lingcod in Alaska is not well-established. In Canada's Queen Charlotte Islands size at which >50% of the males mature ranges between 58 and 62 cm TL. At 4- and 5-years-old, 20% and 70% of these males are mature, respectively [23]. No anal papilla indicative of a mature male was observed in any lingcod <60 cm TL during either fall 2008 or summer 2009 tagging activities. However, based on depth data from lingcod tagged with pressure sensors, we believe that at least one fish, initially tagged at 54 cm TL, exhibited nest guarding behavior during the 2009 breeding season. This same fish dispersed in spring 2009.

Our study confirms that lingcod 2- to 4-years-old will exhibit residency for several months at a time. We documented long maximum residency periods, limited movement between pinnacles, and a relatively small number of temporary absences in non-dispersing lingcod as well as dispersing lingcod prior to their departure. Our results are in contrast to what Starr and others [10] observed in lingcod monitored over a 436 d study in southeast Alaska. There, lingcod resided in the reserve an average of 12.1 d (±1.0 SE) at a time. Tagged lingcod in their study, however, were all adults >80 cm TL. Interestingly, relatively short absences were characteristic for both studies. In southeast Alaska fish remained outside the study area on average 6.5 d, and 50% stayed away <2 d.

The Copper River Delta borders Prince William Sound and in late spring, high densities of lingcod in pursuit of returning adult Chinook (*Oncorhynchus tshawytscha*) and sockeye salmon (*O. nerka*) have been regularly noted by offshore fishers. We had hypothesized that we would detect lingcod movements in Port Gravina coinciding with the seasonal return of salmon and Pacific herring (*Clupea pallasi*), both common prey of lingcod [2]. At the end of March 2009 schools of adult Pacific herring moved into Port Gravina and spawned along both shorelines at and near the mouth of the bay through 9 April. Two of the thirteen monitored lingcod, one from central reef and one from Gravina Rocks were absent at least once for up to 7 d during this period. Interestingly, we did not detect movements that would indicate pursuit of either returning coho *(O. kisutch)* or pink salmon (*O. gorbuscha*) in August and September, despite the presence of two coho spawning streams close to the west side of the Port Gravina curtain as well as a pink salmon spawning stream close to the Gravina Rocks tagging area.

In conclusion, our results provide new insights into the seasonal and ontogenetic influences on lingcod movements. More broadly, our results are the first to correlate individual variability in migration timing with the subadult stage. While this phenomenon is assumed to occur for many species, confirmation has been difficult without the application of acoustic monitoring. The relatively high proportion of fish migrating out of Port Gravina may reflect a high level of connectivity within Prince William Sound and possibly the Gulf of Alaska subpopulations. In the future, the Ocean Tracking Network and the POST Project have proposed to install curtains of receivers across Hinchinbrook Entrance and Montague Strait, two major channels connecting Prince William Sound to the Gulf of Alaska. Future tagging of lingcod in these areas will help to clarify the extent of movements between these two bodies of water.

## Materials and Methods

### Ethics Statement

Capture, handling, and tagging procedures were approved by the University of South Alabama's Institutional Animal Care and Use Committee (IACUC Protocol 05045 issued to Sean P. Powers).

Port Gravina (60°40′N; 146°20′W) is a large bay located in northeast Prince William Sound, Alaska. The Bay is historically an important overwintering and spring spawning area for adult Pacific Herring [24] and supports coho and pink salmon spawning streams. Our study area was the mouth of Port Gravina, with our tagging efforts focused at two areas: 1) Gravina Rocks near the eastern shore; and, 2) central reef, an isolated reef in the center of the Bay's mouth. Gravina Rocks area consists of one supratidal pinnacle and four subtidal pinnacles (10–20 m MLW) with areal surfaces ranging from 0.05 to 0.11 km^2^. The central reef is a single pinnacle rising to 7 m MLW with an areal surface of 0.7 km^2^. Both areas consist of relatively low-relief, mixed substrates composed of rock, sand, and shell. Macrophyte coverage varies from 20–60% and is dominated by *Agarum clathratum* (sieve kelp), and secondarily by *Laminarian* algae. In addition to lingcod, these pinnacles are inhabited by high densities of adult rockfish (*Sebastes spp*).

Twenty-one acoustic receivers (VR series, Vemco, Halifax, Canada) were deployed in October 2008. Depending on the model, receivers were tethered to stationary moorings at depths ranging from 43 to 130 m (VR3) and 7 to 17 m (VR2W). Thirteen receivers were placed 750 m apart to create a “curtain” across the mouth of Port Gravina. One receiver was placed 1.2 km south of the curtain near Gravina Island. At Gravina Rocks and the central reef, five and two receivers, respectively were positioned on the subtidal pinnacles. Based on tag detection range, we estimated receiver coverage at 2.0 km^2^ for Gravina Rocks and 0.7 km^2^ at central reef study areas. Data from receivers were uploaded at least twice per year with the most recent upload 23 February 2010.

Lingcod were captured in and around the subtidal pinnacles using hook and line. Following capture, we placed each fish into a 40 gallon plastic aquarium containing a solution of ambient seawater and clove oil (40 mg/L), an anesthetic. We removed each fish from the solution when it became motionless, placed it on a V-shaped surgery board lined with a clean, disposable plastic surgical mat and pumped seawater through the fish's mouth and out through the opercular cavity. For tag insertion, we made a small incision (2 cm) in the abdominal cavity. A Vemco series V13-1L acoustic transmitter (Vemco, Halifax, Nova Scotia) programmed to transmit an individually-encoded signal at 60–120 s random intervals was placed below the stomach, against the abdominal cavity. Tags equipped with pressure sensors (n = 12) were rated to 200 m depth and measured 45×13 mm with an estimated battery life of 742 d. Tags without pressure sensors (n = 10) measured 36×13 mm and have an estimated battery life of 879 d. The incision was closed with two sutures and swabbed with a broad spectrum antibiotic ointment. Surgery took less than 3 min. We also measured total length (mm) and tagged each fish with an external t-bar tag (46×2 mm) anchored below the dorsal ray. Following surgery, fish were held for recovery in a 40 gallon plastic aquarium with ambient seawater until equilibrium (upright swimming) and active swimming were observed. Recovery was typically observed within 2 to 10 min. Post recovery we released fish in the central part of the acoustic hydrophone array at the capture site.

We determined tag detection range by attaching a V13-1L transmitter to a weighted fishing line and lowering the tag 12 m below the research vessel. We then positioned the vessel directly over a receiver moored at 12 m. The distance between the research vessel and the receiver was then increased by 10 m increments at 3 min intervals. The range test was repeated and the effective detection distance was estimated at 400 m.

Sex was determined by noting the presence of anal papillae, a physical characteristic of mature male lingcod. When a papillae was not apparent, lingcod >600 mm TL were assumed to be females and lingcod <600 mm TL were assigned unknown sex. Age/length data are not well-documented for lingcod in Alaska. Age at capture was estimated using unpublished data provided by Alaska Department of Fish and Game for approximately 500 lingcod ≤6-years-old from southcentral Alaska and aged using dorsal fin rays (Scott Meyer, Dec. 2009, pers. commun.).

In order to determine residency we plotted acoustic receiver detections for each tag by date. We assumed that a fish was resident on days with ≥2 detections at their Gravina Rocks or central reef capture area. For each fish we calculated a percent residency index based on the number of days an individual was resident/length of time an individual was monitored (tag date to date of last detection). We also calculated the maximum number of consecutive days a fish was detected. Movements out of the study area were characterized as temporary (≥1 d absence followed by a return to the study area) or a dispersal (moving out of the tagging site and not returning). Fish were considered having migrated out of Port Gravina if their movements were southward and their final detection was either at the Port Gravina curtain or Gravina Island. Potential mortalities of tagged fish were assessed by analyzing individual fish data for variability in patterns of detection among study area receivers. For example, if a fish was detected solely at one receiver and later solely at another receiver, such a pattern of detection indicated a movement across the area of receiver overlap. Similarly, irregular periods with no detections indicated some degree of movement beyond receiver detection. A regular and non-varying pattern of detection indicated a potential mortality.

Statistical analyses were performed using StatView (SAS Institute, Cary, NC). Significant differences in group means were determined using t-tests. We used analysis of variance (ANOVA) to test for differences in the average duration of absences between individual fish. Significance level for all tests was *P*≤0.05. Data are reported as 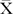
±SE.
